# Introduction to the Research Topic Novel Insights in Rehabilitation of Neglect

**DOI:** 10.3389/fnhum.2014.00233

**Published:** 2014-04-14

**Authors:** Stefan Van der Stigchel, Tanja C. W. Nijboer

**Affiliations:** ^1^Department of Experimental Psychology, Helmholtz Institute, Utrecht University, Utrecht, Netherlands; ^2^Brain Center Rudolf Magnus and Center of Excellence for Rehabilitation Medicine, University Medical Center Utrecht and De Hoogstraat Rehabilitation Center, Utrecht, Netherlands

**Keywords:** neglect, rehabilitation, treatment, prism adaptation, stroke

Hemispatial neglect is the failure to report, respond to, or orient to novel or meaningful stimuli presented in the contralesional visual field. This failure cannot be attributed to motor or sensory defects (Heilman and Valenstein, 1979). It constitutes one of the most invalidating neurological disorders that can occur after stroke. As discussed in this Research Topic, patients with neglect are less independent in various activities of daily living compared to patients without neglect (Nijboer et al., 2013). It is therefore important to treat neglect as adequately as possible and much of the research dedicated to neglect therefore focuses on rehabilitation. Here we provide a brief overview of the 29 articles featured in this Research Topic.

This Research Topic points to a number of promising technological innovations. For instance, it is argued that computer-based testing allows more sensitive quantification of attentional disorders and recovery than paper-and-pencil tests (Bonato and Deouell, 2013). These innovations are likely to result in improved diagnosis and more tailor-made rehabilitation trajectories. Furthermore, future studies will hopefully take into account improved statistical approaches, like mixed linear modeling, which are more appropriate than ANOVAs to assess change over time when measuring recovery patterns (Goedert et al., 2013). Also innovations are proposed with respect to treatment of neglect. Prism adaptation (PA) is currently the most profoundly studied rehabilitation technique for neglect. New insights are reported in this Research Topic. First, the effect of PA extends to walking trajectories: PA when applied to the upper right limb improved the walking trajectory of a neglect patient, and this effect remained up to 15 months after treatment (Rabuffetti et al., 2013). Second, in line with the technological innovations mentioned above, computer-based PA is shown to be feasible, yet no improvement of neglect has been found on neuropsychological neglect tests (Smit et al., 2013). Third, two studies aimed to unravel the specific conditions in which the beneficial effects of PA are optimal. One of the articles discusses an effective novel adaptation procedure, which is more ecologically valid and regarded as more pleasant by patients (Fortis et al., 2013). The success of this new procedure is also highlighted in a review on different PA procedures, which revealed that the different available PA procedures are equally effective (Facchin et al., 2013). The results of these studies indicate that one can choose the best fitting or most suitable procedure for a given patient, without lowering the efficacy of the PA adaptation itself.

The underlying mechanism of PA is currently unclear. In this Research Topic, there was a debate on which aspect of neglect is part of the successful PA treatment: the perceptual or visual aspect or the motor aspect (Saevarsson and Kristjansson, 2013; Striemer and Danckert, 2013), whereas another research proposes that a distortion of visual space explains neglect performance while adapting to prisms (Scriven and Newport, 2013). This debate is still ongoing and will hopefully be resolved in the coming years, perhaps by using relatively novel measures like visually evoked magnetic fields (Mizuno et al., 2013).

Besides PA, a wide range of rehabilitation techniques tapping into various domains underlying hemispatial neglect, such as galvanic vestibular stimulation (Schmidt et al., 2013), transcutaneous electrical nerve stimulation (Pitzalis et al., 2013), motivational manipulations (Russell et al., 2013), visual scanning training (Van Kessel et al., 2013), space- and alertness-related training (Sturm et al., 2013), limb activation training (Pitteri et al., 2013), pro-cholinergic treatments (Lucas et al., 2013), and optokinetic stimulation (Daini et al., 2013), are described. From this list, it becomes clear that there is a wealth of different techniques, although effectiveness was shown to be quite diverse. One study directly compares the beneficial effects of visual scanning training, PA, and limb activation and reveals that all three treatments can be considered as comparably effective rehabilitation interventions (Priftis et al., 2013). There are also newly proposed techniques, such as noradrenergic stimulation to improve motor neglect (Sampanis and Riddoch, 2013) and videogame based neglect rehabilitation due to their high flexibility (Borghese et al., 2013).

Systematic reviews of the different techniques point to major shortcomings of the current literature on rehabilitation methods of neglect. The effectiveness of almost all techniques has not been investigated thoroughly enough to allow firm conclusions (Fasotti and van Kessel, 2013). For instance, when looking at the studies that used the behavioral inattention test as the primary outcome, the conclusion was drawn that all these studies had low power and suffered from limitations in the blinding of the design (Yang et al., 2013). With respect to upcoming non-invasive brain stimulations, such as TMS and tDCS, only few studies are reported, which are too heterogenous in methodology and outcome measures to draw firm conclusions on effectiveness from them (Muri et al., 2013). The same conclusion holds for eye patching, for which there is a great need for randomized controlled trials (Smania et al., 2013).

One of the factors that might contribute to the lack of consistent findings on the different rehabilitation techniques is the heterogeneity of the neglect syndrome. One of the proposals in this Research Topic is that a deficit in spatial working memory is one of the possible components of neglect. With respect to treatment, it is known that for example PA has no influence on spatial working memory deficits, which might explain why some patients benefit from PA whereas others do not (Striemer et al., 2013). Others characterize neglect as a disorder in representational updating, which reflects our ability to build mental models and adapt those models to changing experience (Shaqiri et al., 2013). Furthermore, neglect might be related to the motor system as reflected by a case description of a patient with motor extinction (Punt et al., 2013).

## Conclusion

This Research Topic has opened new perspectives, and has given us an indication of where the field is going. Although some of the current rehabilitation techniques have proven to be beneficial, there is limited agreement on the most valuable technique or the mechanisms underlying the ameliorating effects. Future studies should focus on the heterogeneous nature of the neglect syndrome. There is a need for a better link between the various primary components of neglect and a more sensitive diagnosis (e.g., using computer-based testing) in future rehabilitation studies.

## Conflict of Interest Statement

The authors declare that the research was conducted in the absence of any commercial or financial relationships that could be construed as a potential conflict of interest.
